# Effectiveness of Sacral Epidural Laser Discectomy in Patients with Chronic Low Back Pain Resistant to Conservative Treatment

**DOI:** 10.3390/jcm14176192

**Published:** 2025-09-02

**Authors:** Bora Uzuner, Dursun Türköz, Dilek Durmuş

**Affiliations:** 1 Department of Physical Medicine and Rehabilitation, Pain Management Section, Ondokuz Mayıs University, 55270 Samsun, Turkey; 2 Samsun Eğitim Ve Araştirma Hastanesi, 55090 Samsun, Turkey; turkozdursun@gmail.com; 3 Department of Physical Medicine and Rehabilitation, Medical Faculty, Ondokuz Mayıs University, 55270 Samsun, Turkey; drdilekdurmus@yahoo.com

**Keywords:** chronic low back pain, epiduroscopy, laser discectomy

## Abstract

**Background/Objectives:** This study aimed to evaluate the effect of sacral epidural laser discectomy (SELD) on clinical parameters in patients with chronic low back and/or leg pain (CLBLP) resistant to conservative treatment. **Methods:** A total of 75 patients with CLBLP who received SELD treatment were retrospectively included in this study. Patients were assessed for pain (numeric rating scale—NRS) and disability (Oswestry Disability Index—ODI). NRS and ODI scores were recorded before the operation and 1, 6, and 12 months after the operation. **Results:** Of the 75 patients, with a mean age of 52 ± 11 years (range: 30–78 years), 45 (60.0%) were female and 35 (40.0%) were male. The baseline pain intensity (7.43 ± 0.774) and pain intensities obtained at three time points following the surgeries (1 month [3.93 ± 1.571], 6 months [4.36 ± 1.591], 12 months [5.00 ± 1.716]) showed statistically significant differences (*p* < 0.001). The baseline pain-related disability (2.92 ± 0.539) and the data obtained at three subsequent time points (1 month [1.76 ± 0.883], 6 months [1.85 ± 0.896], and 12 months [2.01 ± 0.923]) showed a statistically significant difference in pain-related disability (*p* < 0.001). The most common complications were headache (five patients) and incisional pain (five patients). **Conclusions:** As a result of this study, we found that SELD reduces pain and disability in patients with conservative-treatment-resistant CLBLP. Although serious complications may rarely occur, the procedure is generally associated with an acceptable and low complication rate.

## 1. Introduction

In the last decade, the human lifespan has significantly increased. However, the quality of life related to health has not kept pace with the lifespan extension, leading to an increase in morbidity. Low back pain is the most common cause of disability worldwide [1]. Unfortunately, the treatments applied for chronic low back pain (CLBP) demonstrate limited effectiveness. The methods most frequently preferred by physicians for treatment include medications, exercise, and manipulation, which generally result in a 10- to 20-point improvement on a 100-point visual analogue scale compared to placebos in large-scale studies. Moreover, even widely used medications such as pregabalin are often prescribed with hesitation due to concerns about safety, addiction risk, and limited effectiveness in chronic pain [2]. For this reason, in many cases, physicians often combine multiple treatment methods with the hope of achieving sufficient analgesic effects. Among the many existing treatment methods, epidural injections are commonly utilized for pathological conditions, such as lumbar disc herniation, spinal stenosis, discogenic pain, and post-laminectomy syndrome [3]. In cases of persistent disc herniation or post-laminectomy syndrome that do not respond to epidural injections, the adhesiolysis procedure is frequently employed [4,5]. The adhesiolysis procedure can be performed using a soft catheter or a flexible epiduroscope. The goal of the adhesiolysis procedure is to release adhesions surrounding the nerve that compress or pull on it. The aim of improving blood circulation around the nerve is to restore the flow of nutrients from the cerebrospinal fluid to the nerve root. However, adhesiolysis using a soft catheter has limitations, including the inability to address pathologies with dense fibrous tissue or protruded disc tissue effectively.

Epiduroscopy is a percutaneous, minimally invasive procedure used for the diagnosis and treatment of lower-back pain. It is based on visualizing the epidural space through the sacral hiatus using a flexible epiduroscope. When necessary, it allows for various treatment options, such as the administration of therapeutic medications, adhesiolysis, and laser decompression of the herniated disc. Over the years, the applications of epiduroscopy have expanded in parallel with technological advancements. In the early 2000s, the technique of sacral epiduroscopic laser decompression (SELD) was developed using fibre-optic flexible cameras, light sources, and laser technology. SELD has found wide application in the diagnosis and treatment of various pathologies, such as adhesions, spinal stenosis, disc herniations, CLBP, and failed back syndrome in the last three decades [6,7,8,9,10,11,12,13].

The possible mechanisms of action of SELD can be explained by the annulus decompression following laser vaporization of the ruptured disc, adhesiolysis of adhesions around the nerve root and surrounding structures, ablation of the sinuvertebral nerve, and removal of inflammatory cells and mediators from the environment through saline irrigation.

The clinical outcomes of SELD have been reported in a limited number of articles [14]. We aimed to review the effects of SELD treatment on both short-term and long-term pain and disability in a population of patients with CLBP (lasting more than 6 months) and/or leg pain who were resistant to conservative treatment and had previously undergone interventional procedures.

## 2. Methods

### 2.1. Participants

This study retrospectively identified patients who underwent SELD operations at the Education and Research Hospital pain medicine clinic between 11 January 2016 and 31 May 2017 (ethical committee approval no: TUEK 31-2019BADK/7-60). An a priori power analysis was conducted to determine the sample size. Assuming a medium effect size (f = 0.25), an alpha level of 0.05, a statistical power of 0.95, four repeated measurements, and a correlation of 0.5 among repeated measures, the power analysis for a repeated measures ANOVA within-subjects design determined that the required sample size was 46 participants. However, to increase the statistical power of this study, additional participants were included.

Patients who had suffered from lower-back and/or leg pain for at least 6 months, as well as had undergone various treatments (non-pharmacological methods [physical therapy modalities, etc.], medical treatments [non-steroidal drugs, muscle relaxants, antidepressants, antiepileptic agents, opioids, etc.], or interventional procedures [spinal–caudal injections, radiofrequency therapies, etc.]) prior to SELD operation but still had complaints and, therefore, had a SELD operation planned as the next step, were retrospectively evaluated. The exclusion criteria were as follows: being under 18 years old; having significant cognitive impairment and/or psychiatric illness that would impede communication; having cancer; having severe spinal stenosis; and refusing to participate in this study.

### 2.2. Preoperative Protocol

Prior to the operation, all patients were provided detailed information about the possible advantages, disadvantages, and complications, and their consent was obtained. Detailed physical examinations were performed, and basic characteristics obtained from spinal magnetic resonance imaging (MRI) were recorded for each patient.

### 2.3. Sociodemographic Form and Scales

The participants were asked to provide information about their age, gender, presence of additional diseases, and pain areas (they were asked to mark the areas on a prepared diagram). Previously applied interventional procedures and surgical methods were checked in the digital health record system, along with patient statements. A numeric rating scale (NRS) was used to evaluate pain intensity. The participants were asked to choose a number that best reflected the intensity of their pain (0 = no pain and 10 = the most severe pain imaginable). Higher scores indicated greater pain intensity. NRS scores were recorded before the operation (baseline pain intensity) and 1, 6, and 12 months after the operation [15].

The Oswestry Disability Index (ODI) was used to measure disability associated with back pain. It assesses how the patient’s back pain affects their daily tasks and activities and consists of 10 items. Each item is scored from 0 to 5, where higher values are associated with greater disability [16]. ODI results were recorded before the operation (baseline disability) and 1, 6, and 12 months after the operation in the study.

### 2.4. Surgical Technique

**Figure 1:**
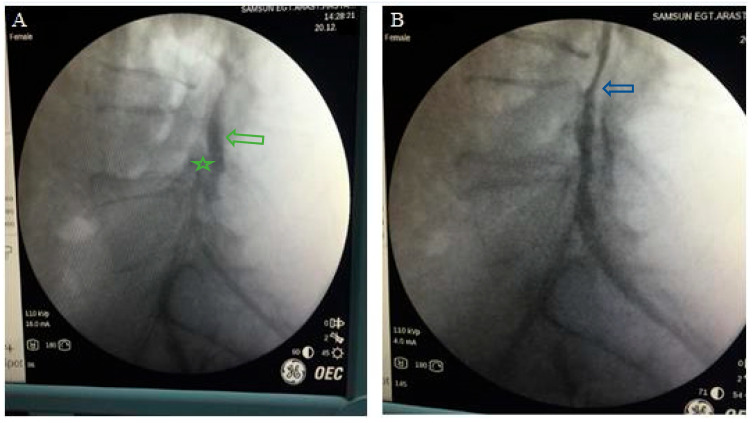
(**A**) A pre-procedure epidurogram obtained before performing SELD highlights the herniated nucleus pulposus and adhesions causing flow obstruction at the affected level. (**B**) A post-procedure epidurogram following SELD demonstrates a reduced herniation outline and restored flow at the previously affected site. (Green star: tip of the catheter (in the anterior epidural space); green arrow: posterior epidural contrast distribution. Blue arrow: spread of contrast medium into the anterior epidural space after adhesiolysis).

The epiduroscopy surgical technique was performed under sterile operating room conditions with hemodynamic and respiratory monitoring, following the administration of preoperative antibiotics. Communication with the patient was maintained during the procedure, and deep sedation was avoided to ensure full neurological monitoring [17]. Before the procedure, the caudal and sacral areas were wiped with an antiseptic solution. Local anesthetics were administered to the skin and subcutaneous tissue. A skin incision was made above the sacral hiatus. A trocar was inserted through the incision site into the sacrum, and C-arm fluoroscopy was used to confirm that the trocar was in the midline with lateral and anteroposterior views. Then, a Spinnaut-V video-guided catheter (IMEDICOM Co., Ltd., Gunpo, Republic of Korea) was inserted into the trocar. The V video-guided catheter was manipulated into the anterior epidural space at the level of S2–S4 vertebrae with a fluoroscopic lateral view. An anterior epidurogram was performed using a non-ionic contrast agent to visualize the dyeing of the anterior epidural space and the outline of its pathology (Figure 1).

**Figure 2:**
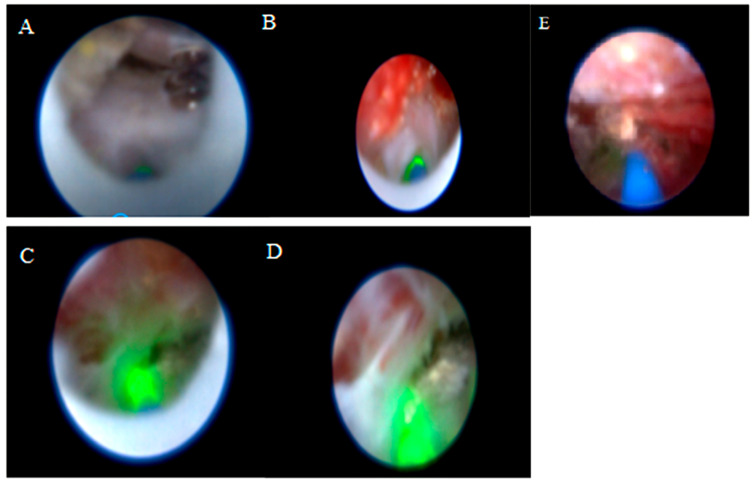
Epiduroscopic visualization: (**A**) The dura is compressed by the protrusion of the posterior longitudinal ligament (PLL), beneath which lies a herniated nucleus pulposus (HNP). (**B**) Decompression of the ruptured HNP located beneath the PLL is achieved using the Ho:YAG laser. (**C**,**D**) Fibrosis and adhesion undergo adhesiolysis with the Ho:YAG laser. (**E**) Increased vascularity.

The vertebral level where the catheter tip was located was determined with fluoroscopy. Mechanical adhesiolysis was performed with a directable video-guided catheter that was washed with saline through the side arm of the cannula for better visualization. The L5-S1, L4-5, and L3-4 levels were viewed using video imaging. Although the procedure is initiated via the sacral hiatus, the flexible video-guided catheter allows advancement cranially up to the L3-L4 level under fluoroscopic guidance. This enables visualization and treatment of upper lumbar disc herniations through the sacral approach. After the herniated disc pathology was observed, a 400 µm diameter Ho: YAG laser probe (ACCU-Tech Co., Ltd., Beijing, China) was advanced to the tip of the video-guided catheter. The lateral view and catheter placement (within the posterior longitudinal ligament [PLL] at the lowest level of the target disc) were confirmed with fluoroscopy and epiduroscopy, aided by video imaging of the anterior epidural space. Using the Ho:YAG laser at 5 W (0.5 J, 10 Hz), a hole was made in the PLL. Then, a fibre video cable was inserted under the herniated intervertebral disc through the hole in the PLL, and the herniated disc was ablated using the Ho:YAG laser at 8 W (0.8 J, 10 Hz). The decompression of the ruptured disc was subsequently confirmed epiduroscopically, demonstrating its decompression from the nerve root (Figure 2). Although standardized laser settings were attempted, some variability occurred because of tissue density and anatomical constraints. At the end of the procedure, 16 mg dexamethasone was applied to the epidural region, and then, the catheter was removed. All procedures were performed by the same clinician, who had at least five years of experience in interventional pain management and had received specialized training in SELD. The patients without any complaints were sent to a postoperative care room [18].

### 2.5. Postoperative Protocol

All major complications occurring during or after the surgery were documented. Surgical outcomes were assessed at the patients’ most recent follow-up using Odom’s criteria. This 4-point grading system evaluates clinical results following spinal surgery as excellent (no symptoms related to lumbar disc disease and no functional limitations), good (occasional discomfort with minimal functional impact), satisfactory (noticeable improvement but with substantial functional restrictions), and poor (no improvement or a worsening of the condition) [19].

### 2.6. Statistical Analysis

Analysis was performed using Statistical Package for Social Sciences (SPSS) version 20 (IBM Corp., Armonk, NY, USA). Continuous variables are expressed as median (min-max), while categorical variables are presented as numbers (%). The normality of the data was tested using the Kolmogorov–Smirnov test. For non-normally distributed data in comparisons between groups, the Kruskal–Wallis analysis of variance test and Mann–Whitney U test were used. The Friedman test was used to compare repeated measurements, and the Wilcoxon signed-rank test was used for pairwise comparisons of significant values. Bonferroni correction was applied for post hoc analyses following comparisons of more than two groups. The chi-square test was used to compare categorical data. A significance level of *p* < 0.050 was considered statistically significant, except for post hoc analyses.

## 3. Results

### 3.1. Patient Characteristics

A total of 106 patients were invited to participate in this study, of whom 85 agreed. Ten patients were lost to follow-up and excluded from the study, resulting in a final sample size of 75 patients. Of the 75 patients who completed the study, with a mean age of 52 ± 11 years (range: 30–78 years), 45 (60.0%) were female and 35 (40.0%) were male. The most common comorbidity among the participants was diabetes mellitus (DM), with 24 (32%) participants reporting this condition. A total of 56 patients (74.7%; 37 [66.1%] female, 19 [33.9%] male) had a history of lumbar surgery.

Pain locations were noted as follows: only in the lower back (without radiating pain) in 8 (10.7%) patients, only in the leg(s) (without back pain) in 22 (29.3%) patients, and both in the lower back and leg(s) in 45 (60.0%) patients. Preoperative MRI examinations of the patients revealed at least one pathological finding (bulging/protrusion/extruded disc herniation, central and/or lateral stenosis, granulation at the surgical site) at the L3-4 level in 17 (22.7%) patients, at the L4-5 level in 59 (78.7%) patients, and at the L5-S1 level in 55 (73.3%) patients. Bulging was observed in 10 (13.3%) patients, protrusion in 60 (80.0%) patients, and extruded disc herniation in 8 (10.7%) patients. Central disc involvement was present in 47 (62.7%) patients, foraminal disc involvement in 28 (37.3%) patients, lumbar stenosis in 43 (57.3%) patients, and granulation in 42 (56.0%) patients. No patients were included without MRI-confirmed pathology; all patients had at least one radiologically significant finding, such as herniation, spinal stenosis, or fibrosis.

Previously performed spinal interventional procedures were as follows: caudal epidural steroid injection (CESI) in 28 patients (37.3%), transforaminal epidural steroid injection (TESI) in 40 patients (53.3%), radiofrequency ablation (RFA) of medial branch nerves for the lumbar spine in 25 patients (33.3%), and dorsal root ganglion (DRG) pulsed radiofrequency (PRF) in 39 patients (52.0%). While 63 individuals (84.0%) underwent one or more of these four procedures, 4 individuals (5.33%) underwent all of them.

### 3.2. Assessment of Pain Intensity and Disability

Pain intensity: Pain intensity was evaluated at four different time points within a one-year period. Baseline pain intensity (7.43 ± 0.774) and pain intensities obtained at three time points following the surgeries (1 month [3.93 ± 1.571], 6 months [4.36 ± 1.591], 12 months [5.00 ± 1.716]) showed statistically significant differences (χ^2^ = 163.39; *p* < 0.001) (Table 1).

**Figure 3:**
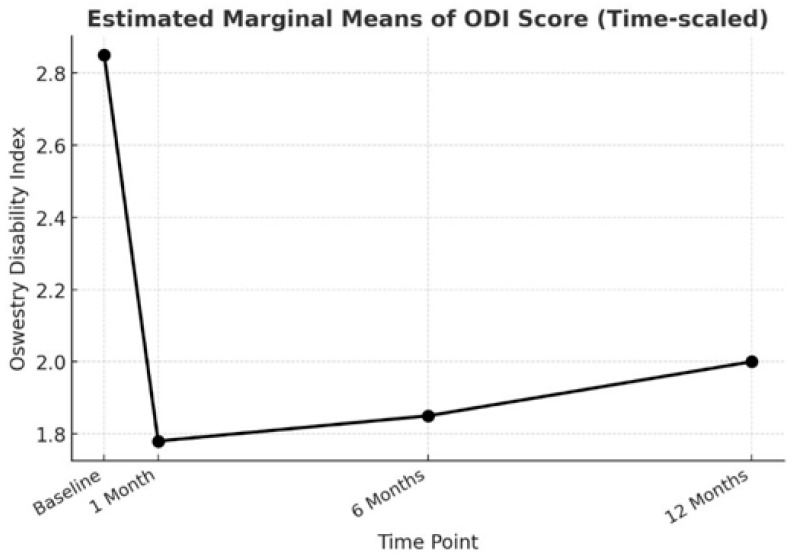
ODI results before (baseline), 1 month, 6 months, and one year after epiduroscopy (χ^2^ = 115.489, *p* < 0.001).

Disability: ODI (Oswestry Disability Index) values were obtained at four different time points within a one-year period. The baseline pain-related disability (2.92 ± 0.539) and the data obtained at three subsequent time points (1 month [1.76 ± 0.883], 6 months [1.85 ± 0.896], 12 months [2.01 ± 0.923]) indicated statistically significant differences in pain-related disability (χ^2^ = 115.489, *p* < 0.001) (Figure 3).

### 3.3. Clinical Outcomes

Clinical outcomes were evaluated according to the Odom criteria. Accordingly, 4 (5.3%) participants achieved “excellent” outcomes, 29 (38.7%) participants achieved “good” outcomes, 27 (36.0%) participants achieved “satisfactory” outcomes, and 15 (20.0%) participants achieved “poor” outcomes. Additionally, 13 (17.3%) participants did not benefit from epiduroscopy and required surgical intervention.

### 3.4. Complications

**Figure 4:**
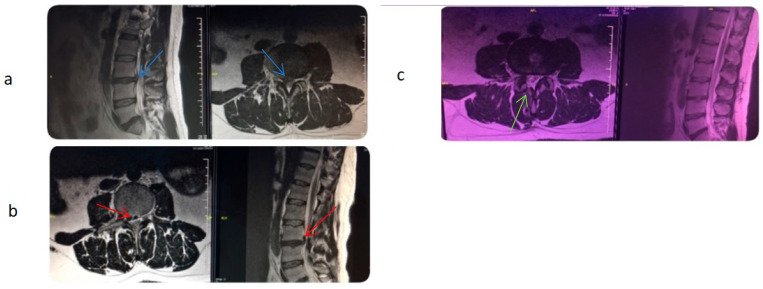
During the SELD procedure performed for a right paramedian disc herniation (blue arrow) at the L3-L4 level (**a**), the patient developed weakness in right knee extension following laser discectomy. Postoperative MRI findings (**b**) revealed cranial and foraminal migration of the paramedian disc (red arrow). The patient underwent a right L3-L4 laminectomy (green arrow) and discectomy one day after the procedure. Postoperative MRI findings following the surgery are shown in (**c**).

The most common complications included headache and incisional pain, each affecting five (6.7%) patients. Other observed complications included dural tear in four (5.3%) patients, motor loss in two (2.7%) patients (one of which was temporary), and infection in one (1.3%) patient. Some individuals experienced multiple complications, with a total of 10 (13.3%) patients developing complications. Among these 10 patients, 8 had a history of lumbar surgery, and 6 had granulation tissue present. One patient who developed motor loss (foot drop) underwent surgical intervention (Figure 4).

### 3.5. Intergroup Comparisons

Demographic data: There was no significant difference in the surgical outcomes (ODOM criteria) between females and males (*p* = 0.566). There was a negative and weak correlation between age and surgical outcomes (r = −0.207; *p* = 0.075). There were no notable differences between the groups concerning the other demographic variables (*p*-values ranged from 0.144 to 0.728).

Pain areas: Surgical outcomes at the 12-month mark were statistically significant in the patient group with leg pain only (without back pain) (χ^2^ = 6.572; *p* = 0.037). No statistically significant differences were identified between the groups regarding pain intensity and pain-related disability across the assessed pain areas (*p*-values: pain intensity = 0.291; disability = 0.215).

Previous surgical history: The patients with and without a history of prior surgery showed no statistically significant differences in pain intensity (*p* = 0.342), pain-related disability (*p* = 0.276), clinical outcomes (*p* = 0.417), or procedure-related complications (*p* = 0.395).

Imaging findings: The patients with and without granulation tissue, spinal stenosis, and various pathologic findings detected on MRI (extrusion, herniation, bulging, etc.) were evaluated separately, and no significant differences were found between the groups (*p*-values ranged from 0.182 to 0.689).

Previous interventional procedures: The types and presence of prior spinal interventional procedures were analyzed, revealing no significant differences between the groups regarding pain (*p* = 0.318), clinical outcomes (*p* = 0.441), or complications (*p* = 0.373).

## 4. Discussion

Lower-back pain is an extremely common health problem affecting individuals of all age groups. In addition to being one of the leading causes of disability, it also causes a significant socioeconomic burden [20].

Most back pain episodes resolve on their own, regardless of treatment, and most individuals with back pain do not pursue medical attention [21]. Approximately 10% to 15% of back pain becomes chronic, and it can cause substantial disability for some in this group. Most patients with low back pain do well with conservative management. However, some patients do not respond to these conservative measures. Many percutaneous disc procedures are in use, and new ones are continually developed to treat patients with discogenic pain that has failed to respond to more conservative management [22]. None of these, however, has been definitively shown to provide better results than a surgical microdiscectomy. SELD is a minimally invasive procedure that allows for direct visualization and treatment of spinal disorders and can be conducted under local anesthesia [11,23]. SELD offers several advantages, including the preservation of paravertebral muscles, protection of bony structures, and rapid recovery. The SELD technique has advanced significantly since Choy et al. [5] first described laser ablation of the intervertebral disc [8,11,12,24,25]. In our study, the long-term (1-year) effectiveness of SELD was retrospectively evaluated in patients with chronic low back pain who were unresponsive to conventional interventional pain treatments, such as transforaminal/interlaminar/caudal epidural steroid injections and percutaneous facet medial branch radiofrequency ablation.

A recently published review reported a clinically significant reduction in pain scores, with an average decrease of 2.8 points and a 20% reduction in disability at 12 months following epiduroscopy treatment. The review suggested that this could be an effective treatment option for patients with failed back surgery syndrome (FBSS) [26]. Similarly, in our study, when we compared baseline pain intensity with postoperative pain intensity at all time points, we found a statistically significant reduction in pain intensity. At 12 months, we calculated an average decrease of 2.43 points in pain intensity. Similarly, for disability, we obtained significant results across all time points and calculated a 31.1% change at 12 months. Most of the patients in our study (74.7%) had a history of prior lumbar surgery, and all patients had previously undergone conventional interventional treatments due to their pain. While SELD yielded favourable outcomes (ODOM 3 and 4) in 44% of the patients, 20% had poor outcomes, and 17.3% required further surgery. Moreover, the rebound in pain and disability scores between 1 and 12 months suggests a potential waning of efficacy over time. We believe that this modest improvement in outcomes is clinically meaningful, particularly for patients unresponsive to conventional interventional pain treatments. Several previous studies showed that the clinical outcomes of SELD are favourable. These studies reported significant reductions in low back pain or radiating leg pain, with patient satisfaction rates exceeding 70%, and a low incidence of failure or recurrence [27,28,29]. The one-year follow-up satisfaction rate observed in our study appears lower compared to these studies. However, Seong Son et al. [24] reported a patient satisfaction rate of 58.5% and a reintervention rate of 17.1% in their retrospective study of 82 patients with a 6-month follow-up period. Their findings are consistent with our results and align with the literature [11,26,29,30,31]. In our study, a slight worsening in pain intensity and disability was observed over time, which is similar to findings reported in a recent study [32]. Although fibrosis recurrence following the epiduroscopy procedure is a significant cause of pain, ongoing pathological degenerative processes in the lumbar vertebrae can lead to the re-release of cytokines, subsequently triggering pain and ultimately resulting in further adhesions.

We could not establish a direct relationship between demographic data and clinical outcomes. We acknowledge that our sample size was not large enough to allow for such comparisons and emphasize the need to evaluate other potential factors that negatively affect chronic pain with a larger cohort. The mean age of the participants was calculated as 52 years, which is consistent with previous studies [31,33]. Compared to those with axial pain, we found significantly better clinical outcomes at the 12-month follow-up in the group with radicular pain. This may be due to the presence of different mechanical causes of axial low back pain in our patients (such as facet arthrosis, instability, spondylolisthesis, or sacroiliac joint-related pain).

The relationship between the type of spinal surgery and epiduroscopy outcomes has been examined in the literature. Compared to discectomy and laminectomy, clinical outcomes were found to be worse in patients undergoing stabilization surgeries and anterior or posterior lumbar interbody fusion [34,35]. However, similar to two separate retrospective studies evaluating the effectiveness of SELD [36,37], we did not find a significant difference in clinical outcomes between patients with or without a history of surgery. The severity of fibrosis may depend on the surgical technique, frequency, and method used [38]. Dense fibrous scar tissue that can form in the epidural space after surgery may cause adhesions on the dura mater and nerve roots, which is associated with FBSS [39,40]. In epiduroscopy, therapeutic efficacy is linked to the endoscopic adhesiolysis of these lesions [26]. Fibrous scar tissue was observed in 42 patients (56.0%); however, we did not find any difference in clinical efficacy between patients with or without fibrosis. Our study included a relatively small patient cohort compared to other studies, which may have prevented some results from reaching statistical significance (e.g., the clinical impact of fibrosis).

We encountered several complications related to epiduroscopy, including transient headaches (*n* = 5), pain at the site of scope insertion (*n* = 5), dural tears (*n* = 4), motor loss (*n* = 2), and mild meningitis (*n* = 1). Except for one patient, all symptoms resolved following bed rest and conservative treatment with medications. A single instance of foot drop due to a laser complication required emergency surgery and was successfully managed. The same patient also developed signs of mild meningitis postoperatively and was treated with intravenous antibiotics during hospitalization. It could not be determined whether the meningitis occurred following the epiduroscopy or the subsequent laminectomy. The patient made a full recovery after completing antibiotic therapy.

Various complications, such as postoperative headache, pain at the incision site, motor loss, and infection, have been reported with SELD [11], and the “60-limit rule” is optionally recommended for these complications [41]. We performed our procedures in accordance with this recommendation. The most common complications included headache and incisional pain, each affecting five patients. Among the major complications, dural tears occurred in four (5.3%) of our patients. Dural tear rates associated with SELD were documented in the literature to range from 1.7% to 7% [7,37]. In patients with a history of previous surgery, the incidence of dural puncture is notably higher compared to those without a surgical history. This increased rate may be associated with anatomical changes and adhesions resulting from prior surgeries, which complicate catheter guidance [41]. In our patient population, 74.7% had a prior surgical history, which may explain the relatively high rate of dural puncture. Notably, in patients who undergo epiduroscopy with laser application, a significant complication arises from thermal damage to the nerve roots, leading to motor nerve dysfunction. Therefore, special attention is required during the laser procedure [9]. In our patient series, motor loss was observed in two patients following laser discectomy, and one of them required emergency surgery.

This study has several limitations that should be considered when interpreting the results. First, the data were collected retrospectively, and the study was designed as a single-arm analysis without a control group (e.g., sham procedure or conventional discectomy). This design inherently limits causal inference and may lead to an overestimation of the treatment’s effectiveness. Second, this study was conducted at a single centre, which restricts the generalizability of the findings and prevents comparisons with populations from different socioeconomic and cultural backgrounds. Third, although SELD is a technically standardized procedure, variations in operator experience and laser settings were not formally controlled. Fourth, sufficient information regarding pain-related emotional and cognitive processes—such as depression, anxiety, or pain catastrophizing—that might influence clinical outcomes was not collected. Finally, no intention-to-treat analysis was performed, and 10 out of the 85 enrolled patients were lost to follow-up, introducing potential attrition bias. Further prospective, controlled studies with larger and more diverse populations are needed to confirm these findings and better delineate the factors influencing SELD outcomes.

## 5. Conclusions

SELD is a minimally invasive technique that has been in clinical use for over two decades to treat patients with back and/or leg pain. It is an advanced method with a low complication rate, but it has the potential for serious adverse events. When performed by experienced practitioners and applied in appropriate indications, it demonstrates notable clinical efficacy. However, most studies in the medical literature are retrospective, including our study. We suggest conducting prospective studies with larger patient cohorts to evaluate the long-term effectiveness of SELD and to assess its associated complications.

## Figures and Tables

**Table 1:** Pairwise comparisons of pain intensity and pain disability.

	Baseline—1. m	Baseline—6. m	Baseline—1. y	1. m—6. m	1. m—1. y	6. m—1. y
Pain intensity *						
Z	−7.380 ^a^	−7.224 ^a^	−6.616 ^a^	−2.542 ^b^	−4.867 ^b^	−4.607 ^b^
Asymp. Sig. (two-tailed)	<0.001	<0.001	<0.001	0.011	<0.001	<0.001
Pain disability **						
Z	−6.610 ^a^	−6.373 ^a^	−5.930 ^a^	−1.400 ^b^	−3.043 ^b^	−3.207 ^b^
Asymp. Sig. (two-tailed)	<0.001	<0.001	<0.001	0.162	0.002	<0.001

Abbreviations: m, month; y, year. Wilcoxon signed-rank test. ^a^: Based on positive ranks. ^b^: Based on negative ranks. *: Pain intensity was calculated using an NRS. **: Pain-related disability was calculated using the ODI.

## Data Availability

The raw data supporting the conclusions of this article will be made available by the authors on request.
